# *Saccharomyces* Genome Database: Advances in Genome Annotation, Expanded Biochemical Pathways, and Other Key Enhancements

**DOI:** 10.1101/2024.09.16.613348

**Published:** 2024-09-20

**Authors:** Stacia R. Engel, Suzi Aleksander, Robert S. Nash, Edith D. Wong, Shuai Weng, Stuart R. Miyasato, Gavin Sherlock, J. Michael Cherry

**Affiliations:** Department of Genetics, Stanford University, Palo Alto, CA 94304, USA

## Abstract

Budding yeast (*Saccharomyces cerevisiae*) is the most extensively characterized eukaryotic model organism and has long been used to gain insight into the fundamentals of genetics, cellular biology, and the functions of specific genes and proteins. The *Saccharomyces* Genome Database (SGD) is a scientific resource that provides information about the genome and biology of *S. cerevisiae*. For more than 30 years, SGD has maintained the genetic nomenclature, chromosome maps, and functional annotation for budding yeast along with search and analysis tools to explore these data. Here we describe recent updates at SGD, including the two most recent reference genome annotation updates, expanded biochemical pathways representation, changes to SGD search and data files, and other enhancements to the SGD website and user interface. These activities are part of our continuing effort to promote insights gained from yeast to enable the discovery of functional relationships between sequence and gene products in fungi and higher eukaryotes.

## INTRODUCTION

The *Saccharomyces* Genome Database (SGD; https://www.yeastgenome.org) is a scientific knowledgebase that provides comprehensive and up-to-date information about the genome and biology of the yeast *Saccharomyces cerevisiae*. It serves as a valuable resource for researchers studying yeast biology and genetics by offering information on genes, proteins, pathways, and phenotypes. Scientists can use SGD to explore the functions of genes, track genetic and physical interactions, and access curated literature related to yeast genetics and genomics. SGD plays a crucial role in advancing our understanding of molecular mechanisms and processes in yeast and serves as a central repository for yeast-related data and information.

Since 1993, SGD has been assembling and cataloging scientific data regarding the genome and proteome of budding yeast, and distributing that information to the public via an open-access web interface and download service. Budding yeast data that can be found at SGD include the reference genome (Engel et al. 2022), which is a single consensus representative *S. cerevisiae* genome sequence against which all other sequences can be compared, and various analysis tools (Balakrishnan et al. 2004, Cherry 2015, Christie et al. 2004, Hirschman et al. 2006, Sheppard et al. 2016) and data files that allow interrogation of the genome sequence and its products for a wide variety of applications (Engel et al. 2018, Hellerstedt et al. 2017, Ng et al. 2019, Wong et al. 2019).

Yeast have proven especially useful for studying various aspects of biology, including the regulation of gene expression through upstream open reading frames (uORFs; Blank et al. 2017, Cartwright et al. 2017, Vindu et al. 2021, Yang et al. 2023), emergence of newly evolved genes (Chang et al. 2023, Wacholder and Carvunis 2023, Wacholder et al. 2023), complex gene structure (Balarezo-Cisneros et al. 2021, Feng et al. 2022, Xu et al. 2009, Yang et al. 2023), and cellular metabolism (Ljungdahl and Daignan-Fornier 2012, Thomas and Surdin-Kerjan 1997). The conservation of many molecular mechanisms between yeast and higher eukaryotes makes findings from yeast studies broadly applicable to understanding the biology of more complex organisms, including humans. Studying these processes in yeast can help uncover basic biological principles that are widely applicable, both to general understanding and for solutions to specific questions. Findings from yeast studies can be validated in other model organisms or human cell lines to assess conservation and relevance across species.

With its simple and well-characterized genome, *S. cerevisiae* makes it easier to study gene functions, regulatory elements, and cellular processes. Advances in yeast research, such as the development of molecular biology techniques, genetic tools, and resources like SGD make yeast a valuable model organism for transferring knowledge to other species by providing a simpler and more tractable system to study fundamental biological processes, test hypotheses, and develop experimental techniques. Here we describe recent changes at SGD, including the two most recent reference genome annotation updates, expanded biochemical pathways representation, improvements to SGD search and data files, and other enhancements to the SGD website and user interface.

## GENOME ANNOTATION UPDATES

### Genome version R64.4.1

In the first of two recent updates, the *S. cerevisiae* strain S288C reference genome annotation was updated to release R64.4.1, dated 2023-08-23 (Table 1). The underlying genome sequence itself was not altered in any way. The update included the addition of eight noncoding RNAs (ncRNAs) and the addition of three new upstream ORFs (uORFs). Three open reading frames (ORFs) were demoted from ‘Uncharacterized’ to ‘Dubious’ because they were found to overlap tRNAs, have multiple frameshifts and/or indels in the coding region, and had minimal evidence to support their existence.

### Genome version R64.5.1

In the second of two recent updates, the *S. cerevisiae* strain S288C reference genome annotation was updated to release R64.5.1, dated 2024-05-29 (Table 2). Once again, the underlying genome sequence was not altered; the chromosome sequences remain stable and unchanged. The update included the addition of six new ORFs and six new uORFs, a shifted start for one ORF, and the upgrade of one ORF from Dubious to Verified because a stable translation product was detected.

## CHANGES TO *SACCHAROMYCES CEREVISIAE* GFF FILE

The saccharomyces_cerevisiae.gff contains data regarding sequence features of *S. cerevisiae* strain S288C and related information such as locus descriptions and Gene Ontology (The Gene Ontology Consortium 2023) annotations. It is fully compliant with Generic Feature Format Version 3 (https://gmod.org/wiki/GFF3.html), and is updated weekly. This is a standard format used by many genomics and database groups. SGD uses the GFF file to load the reference data tracks into SGD’s genome browser resource (https://jbrowse.yeastgenome.org).

After November 2020, SGD updated the transcript features in the GFF file to reflect experimentally determined transcripts (Pelechano et al. 2013, Ng et al. 2020), when possible. The longest transcripts were determined for two different widely-used growth media - galactose and dextrose. When available, experimentally determined transcripts for one or both conditions were added for a gene. Where these data were absent, transcript entries matching the start and stop coordinates of the ORF were used.

In February 2024, SGD edited the ‘gene’ entries in the file to extend the coordinates to encompass the start and stop coordinates of the longest experimentally determined transcripts, regardless of condition. This change was made in order to comply with JBrowse 2 (Diesh et al. 2023), a newer and more extensible genome browser, which requires that ‘gene’ features in GFF files represent a longer region than the features that make up a ‘gene’ (coding sequences, mRNA, etc.).

## BIOCHEMICAL PATHWAYS

SGD’s YeastPathways (https://pathway.yeastgenome.org; Cherry 2015) is a database of 220 conserved metabolic pathways and their corresponding enzymes in *S. cerevisiae*, manually curated and maintained by the curation team at SGD. YeastPathways enables visualization of yeast metabolism from large metabolic networks to individual pathways, and from biochemical reactions down to individual metabolites. Search tools and click-to-browse features in YeastPathways enable quick navigation and intuitive exploration of yeast metabolism.

We recently completed a major update to the YeastPathways content. As the first major update since 2012, we updated 62 pathways with expert summaries on pathway genetics, biochemistry, regulation, and more. Thirty-three new pathways with specificity for yeast biochemistry were propagated from MetaCyc at SRI (Caspi R, et al. 2018), and 105 existing pathways were edited for proper enzymatic classification, reaction connectivity, and gene attribution. Compounds that were previously missing a chemical structure have also now been updated, along with the stoichiometry and scheme of many pathway reactions.

Because many fundamental molecular processes and pathways are evolutionarily conserved between yeast and higher eukaryotes, including humans, the curated metabolic pathway information has great value for the transfer of knowledge to other organisms. Therefore, the YeastPathways data were exported in BioPAX (Demir et al. 2010) format for import into Noctua, a tool for collaborative curation of biological pathways and gene annotations that was developed by the GO Consortium (Thomas et al. 2019). BioPAX provides a standardized format for representing biological pathways, allowing researchers to integrate pathway information from different sources and databases. Noctua can import pathway data encoded in BioPAX format to populate the pathway editor with molecular interactions, biological processes, and regulatory relationships, and can utilize BioPAX files to combine pathway data from multiple datasets for pathway curation and analysis. Pathways curated and edited in Noctua can be exported both as GO annotations for yeast and orthologous genes in other species, or as pathway annotations in BioPAX, which facilitates sharing of curated pathways with other researchers, databases, and pathway analysis tools using a standard format, promoting data exchange and collaboration within the scientific community.

YeastPathways can be accessed via the Function menu in the purple toolbar that runs across the top of most SGD webpages or from the Pathways section on SGD Gene pages. To make the pathways more readily accessible, we added the pathways to SGD Search. The category “Biochemical Pathways” is now available, with facets (i.e., subcategories) for References and Loci. For even easier access, we also added the Pathway names and IDs to the autocomplete in the Search box, to enable quick browsing.

## UPDATES TO SGD SEARCH

Because utilizing the SGD search box provides the most efficient and direct access to the content on the site, we have recently added new data and modified existing data mappings to optimize search performance and capabilities. We have added a new category for Datasets, with over 3,700 yeast datasets accessible for searching by reference, keyword, assay, and lab. A new Strains subcategory has been added to the Reference search. Macromolecular complexes can now be searched with aliases, reference, subunit, function, process, and location. Alleles can be searched via their descriptions, SGDIDs, reference, allele type, gene, and phenotype. RNA products can now be searched using RNAcentral IDs. The improved search functionality enhances the user experience and increases user satisfaction through improved navigation which provides easier access to information, higher relevance in search results, improved data retrieval, and overall better efficiency.

## OTHER UPDATES TO THE WEB INTERFACE

We regularly update the SGD web interface to enhance user experience, improve visual appeal, incorporate new features, align with modern design trends, increase usability, and improve user engagement. The modifications and enhancements described below make the website more user-friendly and effective without the implementation of major overhauls or revisions.

SGD biocurators use the Chemical Entities of Biological Interest (ChEBI) Ontology (Hastings et al. 2016), maintained by EMBL-EBI, to describe chemicals used in experiments curated from yeast publications and displayed on SGD webpages. We recently added chemical structures provided by ChEBI to the Chemical pages in SGD.

In 2011, SGD implemented InterMine (http://www.InterMine.org; Smith et al. 2012), an open source data warehouse system with a sophisticated querying interface, to create YeastMine (Balakrishnan et al. 2012, Hellerstedt et al. 2017), a multifaceted search and retrieval environment that provided access to diverse data types. YeastMine served as a powerful search interface, a discovery tool, a curation aid, and a complex database presentation format. We recently moved the YeastMine data into AllianceMine, hosted by the Alliance of Genome Resources (Alliance of Genome Resources Consortium 2024), of which SGD is a founding member. Users can get started with AllianceMine by going to the Templates page, and filtering by the category ‘YeastMine’. The data from YeastMine are also available on the SGD Downloads site (http://sgd-archive.yeastgenome.org). Information regarding genes and IDs, etc., are in the chromosomal_features directory, and a variety of annotation files for different types of data can be found in the literature directory.

The implementation of Textpresso (Müller et al. 2004) by SGD has recently been updated. Each week, SGD biocurators triage new publications from PubMed to load the newest yeast papers into the database. Once they have been added into SGD, those papers get indexed and loaded into Textpresso, a tool for full-text mining and searching, which provides results shown in the context of the full text, with matches to query terms highlighted *in situ*. Textpresso allows several user-friendly options, including use of Boolean operators, custom corpus creation allowing users to decide which papers to search, search scope options for document or sentence, and search location options for constraining searches to specific sections of papers. Content updates in SGD’s Textpresso are now happening on a weekly basis, enabling full-text search of the very latest yeast papers added to SGD. Textpresso can be accessed via the “Full-text Search” link under “Literature” in the purple toolbar that runs across the top of most SGD webpages.

SGD was recently chosen as a Global Core Biodata Resource (GCBR; https://globalbiodata.org/what-we-do/global-core-biodata-resources) in recognition of our commitment to providing high-quality and valuable biological data to the global research community. We are honored to be selected as a GCBR, and we are dedicated to upholding the highest standards of data integrity, accessibility, and usability to support cutting-edge research and scientific discovery on a global scale. This recognition motivates us to continue expanding and improving SGD to empower researchers worldwide in advancing knowledge and innovation in yeast genetics, genomics, and the life sciences as a whole.

## FUTURE DIRECTIONS

SGD plays a crucial role in organizing, curating, and disseminating biological information related to the model organism budding yeast *S. cerevisiae*. Because many fundamental molecular processes and pathways are evolutionarily conserved between yeast and higher eukaryotes, *S. cerevisiae* is highly useful for transferring that knowledge to other organisms. As one of seven founders of the Alliance of Genome Resources (Alliance of Genome References Consortium 2024), a new central knowledgebase for *Saccharomyces cerevisiae* (yeast), *Caenorhabditis elegans* (worm), *Drosophila melanogaster* (fly), *Danio rerio* (zebrafish), *Xenopus laevis* (frog), *Rattus norvegicus* (rat), *Mus musculus* (mouse), and *Homo sapiens* (human), SGD is positioned to continue advancing scientific research and supporting the needs of the scientific community. Adopting and promoting data standards and interoperable formats will facilitate data exchange and integration between different model organism databases and biological resources. Ensuring data consistency and compatibility enables seamless collaboration and cross-referencing of information across research communities.

As such, we will continue our work with this consortium to harmonize common data types and create a unified web resource. Integrating data from various sources allows researchers to explore complex biological relationships and gain comprehensive insights into gene function and regulation. A large amount of this work has been completed, and integration proceeds apace. SGD’s JBrowse genome browser, YeastMine data warehouse, and Textpresso full-text search tool have already been incorporated into the Alliance of Genome Resources. Current efforts include an integrated BLAST tool based on SequenceServer (https://sequenceserver.com), which we hope to release later this year.

## Figures and Tables

**Figure 1.**
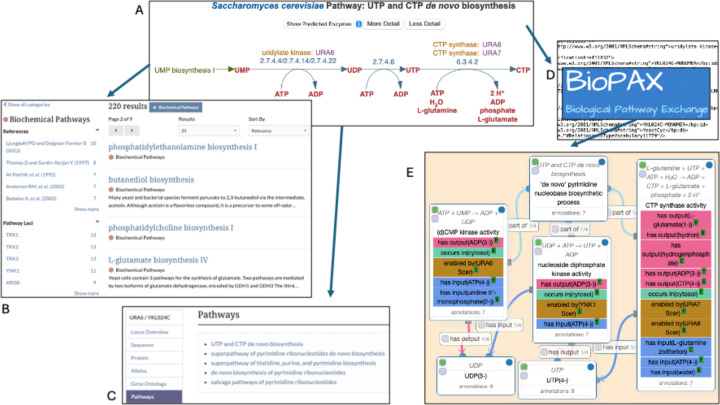
Curated metabolic pathways from YeastPathways (A; UTP and CTP *de novo* biosynthesis, https://pathway.yeastgenome.org/YEAST/NEW-IMAGE?type=PATHWAY&object=PWY-7176) are accessible via SGD Search (B; https://www.yeastgenome.org/search) and SGD Gene pages (C; *URA6*, https://www.yeastgenome.org/locus/URA6). Data from YeastPathways have been exported via BioPAX format (D) to create Gene Ontology annotations using the Noctua collaborative curation tool for pathways and gene annotations (E; UTP and CTP *de novo* biosynthesis, http://noctua.geneontology.org/editor/graph/gomodel:YeastPathways_PWY-7176).

**Table 1.** The *S. cerevisiae* strain S288C reference genome annotation was updated to release R64.4.1, dated 2023-08-23.

Chromosome	Feature	Description of change	Reference
III	*SUT035* / YNCC0015W	New ncRNA chrIII:205766‥205942	Xu et al. 2009, Balarezo-Cisneros et al. 2021
IV	YDR278C	Change ORF qualifier from Uncharacterized to Dubious	Requested by NCBI
IV	*SUT053* / YNCD0033W	New ncRNA chrIV:506334‥507774	Xu et al. 2009, Balarezo-Cisneros et al. 2021
IV	*SUT468* / YNCD0034C	New ncRNA chrIV:506546‥507450	Xu et al. 2009, Balarezo-Cisneros et al. 2021
VII	*SUT532* / YNCG0047C	New ncRNA chrVII:17213‥17709	Xu et al. 2009, Balarezo-Cisneros et al. 2021
VII	*SUT125* / YNCG0048W	New ncRNA chrVII:650855‥651159	Xu et al. 2009, Balarezo-Cisneros et al. 2021, Feng et al. 2022
VII	*SUT126* / YNCG0049W	New ncRNA chrVII:660087‥661399	Xu et al. 2009, Balarezo-Cisneros et al. 2021
XII	*FPS1* / YLL043W	New uORF chrXII:49924‥49932	Cartwright et al. 2017
XIV	*ACC1* / YNR016C	New uORF chrXIV:661704‥661715	Blank et al. 2017
XIV	*HOL1* / YNR055C	New uORF chrXIV:730381‥730401	Vindu et al. 2021
XV	YOL013W-A	Change ORF qualifier from Uncharacterized to Dubious	Requested by NCBI
XVI	*SUT390* / YNCP0025W	New ncRNA chrXVI:52977‥53465	Xu et al. 2009, Feng et al. 2022
XVI	*SUT418* / YNCP0026W	New ncRNA chrXVI:588998‥589830	Xu et al. 2009, Feng et al. 2022
XVI	YPR108W-A	Change ORF qualifier from Uncharacterized to Dubious	Requested by NCBI

**Table 2.** The *S. cerevisiae* strain S288C reference genome annotation was updated to release R64.5.1, dated 2024-05-29.

Chromosome	Feature	Description of change	Reference
II	*ATG12* / YBR217W	New uORF chrII:657824‥657835, partially overlaps CDS	Yang et al. 2023
IV	YDL204W-A	New ORF chrIV:94133‥94285	Wacholder et al. 2023
VI	YFR035W-A	New ORF chrVI:226260‥226550	Wacholder and Carvunis 2023
VII	YGR016C-A	New ORF chrVII:523353‥523246	Wacholder et al. 2023, Chang et al. 2023
IX	*EFM4* / YIL064W	Move start 84 nucleotides downstream, new coordinates chrIX:242027‥242716	Hamey et al. 2024
IX	YIL059C	Change ORF qualifier from Dubious to Verified	Wacholder and Carvunis 2023
XIII	YMR106W-A	New ORF chrXIII:480924‥481187	Wacholder and Carvunis 2023
XIV	YNL040C-A	New ORF chrXIV:552558‥552478	Wacholder et al. 2023
XIV	YNL155C-A	New ORF chrXIV:342135‥341911	Wacholder and Carvunis 2023
XV	*ATG19* / YOL082W	New uORF chrXV:168632‥168679	Yang et al. 2023
XVI	*ATG5* / YPL149W	4 new uORFs: chrXVI:271236‥271277, chrXVI:271252‥271302, chrXVI:271299‥271307, chrXVI:271302‥271307	Yang et al. 2023
XVI	*ATG13* / YPR185W	New uORF chrXVI:907211‥907351, partially overlaps CDS	Yang et al. 2023

## Data Availability

All SGD data and tools are freely available at https://www.yeastgenome.org. The SGD API is freely available at https://www.yeastgenome.org/api/doc. SGD downloads are freely available at http://sgd-archive.yeastgenome.org. YeastMine data within AllianceMine are freely available at https://www.alliancegenome.org/alliancemine.
